# Data in intelligent approach for estimation of disc cutter life using hybrid metaheuristic algorithm

**DOI:** 10.1016/j.dib.2020.106479

**Published:** 2020-11-01

**Authors:** Khalid Elbaz, Shui-Long Shen, Annan Zhou, Zhen-Yu Yin, Hai-Min Lyu

**Affiliations:** a MOE Key Laboratory of Intelligence Manufacturing Technology, Department of Civil and Environmental Engineering, College of Engineering, Shantou University, Guangdong 515063, China; b Discipline of Civil and Infrastructure, School of Engineering, Royal Melbourne Institute of Technology, Victoria 3001, Australia; c Department of Civil and Environmental Engineering, The Hong Kong Polytechnic University, Hung Hom, Kowloon, Hong Kong; d State Key Laboratory of Internet of Things for Smart City and Department of Civil and Environmental Engineering, University of Macau, Macau, China

**Keywords:** Disc cutter, GMDH-type neural network, Tunnel boring machine, Genetic algorithm

## Abstract

This data in brief presents the monitoring data measured during shield tunnelling of Guangzhou–Shenzhen intercity railway project. The monitoring data includes shield operational parameters, geological conditions, and geometry at the site. The presented data were arbitrarily split into two subsets including the training and testing datasets. The field observations are compared to the forecasting values of the disc cutter life assessed using a hybrid metaheuristic algorithm proposed for “Prediction of disc cutter life during shield tunnelling with artificial intelligent via incorporation of genetic algorithm into GMDH-type neural network” [1]. The presented data can provide a guidance for cutter exchange in shield tunnelling.

## Specifications Table

**Table:** 

Subject area	Civil and Environmental Engineering
Specific subject area	Safety, Risk, Tunnelling, Manufacturing, Cost
Type of data	Tables Figures
How data was acquired	Data were recorded by the monitoring systems in the shield machine and the other geological data were measured by surveying
Data format	Raw and analyzed
Parameters for data collection	Monitoring data from the site were collected using borehole samples.
Description of data collection	The laboratory tests such as plasticity index and consistency index of the soil samples were necessary to determine the value of the different variables.
Data source location	Shenzhen, China
Data accessibility	Data provided in the article are accessible to the public. The relevant raw data can be found in this article (see Tables 1 & 2).
Related research article	Elbaz, K., Shen, S.L., Zhou, A.N., Yin, Z.Y., Lyu, H.M. (2020). Prediction of disc cutter life during shield tunnelling with AI via incorporation of genetic algorithm into GMDH-type neural network. Engineering https://doi.org/10.1016/j.eng.2020.02.016

## Value of the Data

• The data is helpful for making a comparison with other artificial intelligent models with high computational performance. Similarly, a benchmark can be made to validate empirical equations and numerical models.
• The data is useful to other scholars who focus on designing and modelling the disc cutter for practical tunnelling applications. Using this data, researchers can assess the behavior of disc cutter during tunnelling especially in rock-soil strata.
• Helpful insights can be gleaned from this data. According to this data, other data can be done that would lead to suitable survey studies.

## 1 Data Description

**Fig. 1:**
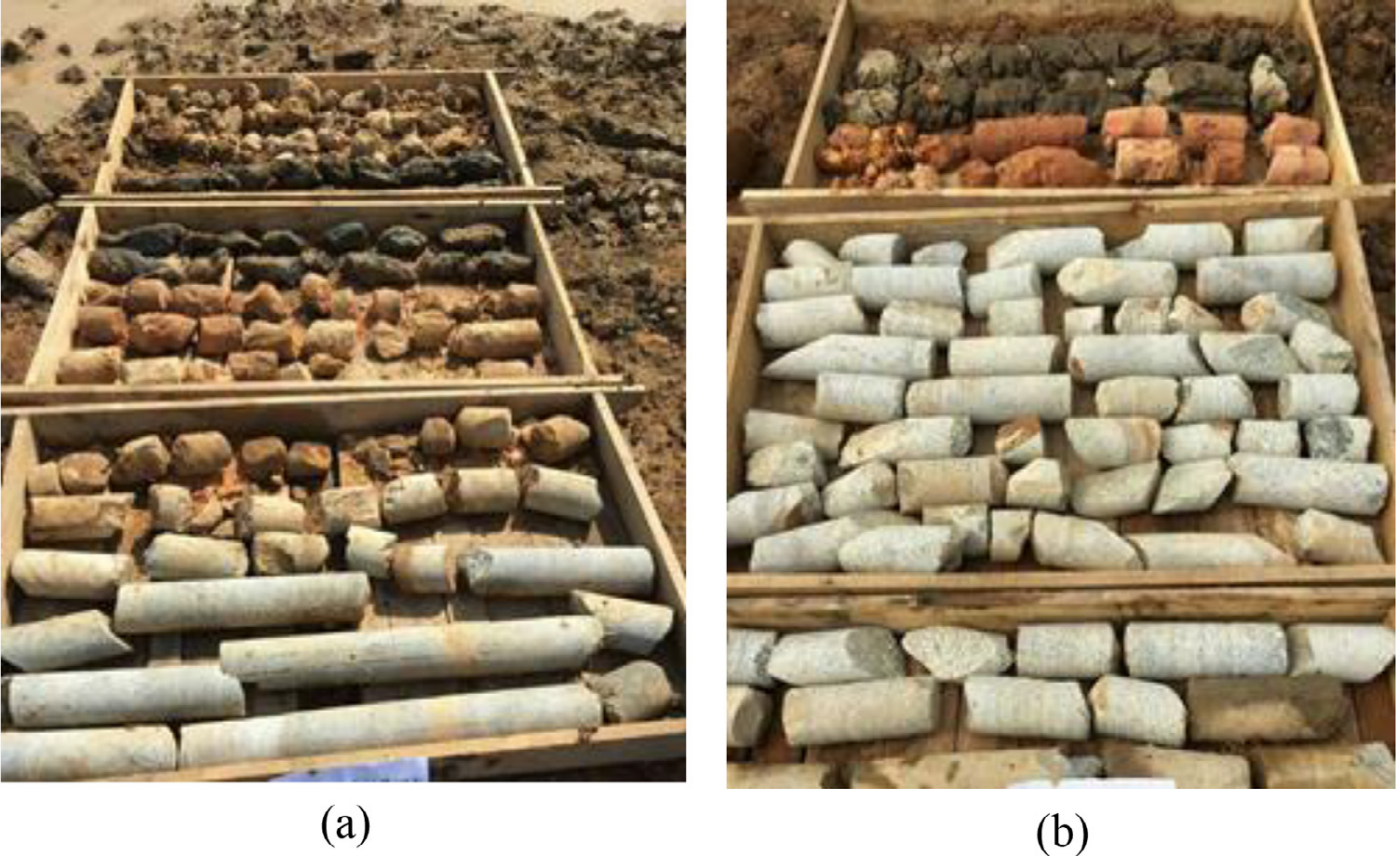
An example of the geological borehole extracted from construction site; (a) Lot ET-3; (b) Lot ET-4.

The database in this article includes the shield operational parameters, geological conditions, and soil geometry. The operational parameters are initially extracted directly from a built-in data acquisition system in the tunnel boring machine. In this paper, geological as-built maps and geological engineering and geotechnical reports from boreholes (Fig. 1) and surface outcrops were considered as sources of geotechnical information for the database [2]. Table 1 lists the data source of the utilized parameters in this paper. The observed data includes operational parameters and geological conditions such as thrust force (TF), cutter rotation speed (RPM), penetration rate (PR), screw rate (SC), grouting pressure (GP), soil pressure (SP), and specific energy (SE), quartz content (Q_c_), excavation depth (H), and disc cutter life (H_f_). The overburden layers of the tunnel were backfill with a thickness about 6 m, clay soil with a thickness of 3.1-8.25 m, silt clay soil with a thickness reaches to 8.5 m.

**Table 1:** Descriptive statistics for average values of the generated database.

No.	H_f_ (m^3^/cutter)	TF (kN)	RPM (rev/min)	SR (rev/min)	PR (mm/rev)	GP (kPa)	SP (kPa)	SE (kWh/m^3^)	Qc (%)	H (m)
1	2700	22000	1.5	11.2	30.00	400	150	2.77017	3.1	14.00
2	2600	23900	1.6	12.2	36.20	400	150	1.67650	3.1	14.30
3	2500	26500	1.6	13.8	26.40	240	170	3.70806	4.5	14.60
4	2250	27000	1.6	15.6	32.00	300	170	2.35586	5.0	14.90
5	1100	29700	1.6	11.0	30.00	340	170	3.12804	6.9	15.80
6	1500	27600	1.6	11.6	35.00	320	160	2.74115	6.9	15.50
7	1800	28700	1.6	14.2	28.00	240	170	7.32218	0.0	15.20
8	1500	26900	1.6	15.2	28.00	340	170	4.15332	7.2	18.78
9	1550	26900	1.6	4.2	28.20	360	160	2.77017	14.0	18.38
10	1430	30000	1.5	10.0	28.80	340	170	3.25733	16.0	19.18
11	1150	40700	1.7	12.4	28.00	300	180	3.25733	0.1	18.50
12	830	32500	1.8	10.6	28.00	320	180	3.10281	4.2	17.60
13	820	36600	1.8	15.6	28.60	300	170	3.25733	4.2	17.30
14	920	37300	1.8	13.2	28.00	310	170	1.84764	13.7	17.90
15	1050	35000	1.8	11.8	31.50	310	170	3.25733	0.0	18.20
16	600	36100	1.8	12.8	28.50	300	190	2.45324	22.0	16.40
17	750	29300	1.8	11.4	30.13	340	200	3.25733	22.0	16.10
18	720	30700	1.8	9.6	18.00	290	220	2.61747	10.3	16.70
19	750	30300	1.9	14.3	25.00	320	220	2.70366	12.1	17.00
20	1200	35500	1.8	11.0	28.00	330	240	2.59635	13.7	18.80
21	1250	38300	1.9	7.0	30.70	320	240	2.70366	22.0	19.10
22	1290	38100	1.9	14.2	30.30	450	240	3.05295	17.6	19.40
23	1330	36600	1.9	12.6	30.80	350	230	3.05295	17.6	19.70
24	1380	35200	1.9	8.2	30.30	310	220	3.25733	14.5	20.00
25	1400	25500	1.8	11.6	28.80	320	220	3.20499	16.0	19.58
26	1600	25500	1.7	7.8	24.00	350	200	4.50374	12.0	17.98
27	1650	21800	1.7	7.6	24.00	330	210	4.50374	12.0	17.58
28	1690	27000	1.7	11.6	23.10	340	200	4.84432	10.1	17.18
29	1800	21700	1.7	12.4	23.70	360	200	4.61452	3.5	15.98
30	1750	28700	1.7	7.0	23.10	340	200	4.84432	3.5	16.78
31	1780	26500	1.7	11.0	23.80	450	180	4.57729	0.9	16.38
32	1830	23300	1.7	14.0	20.50	350	180	5.97992	4.0	15.30

**Fig. 2:**
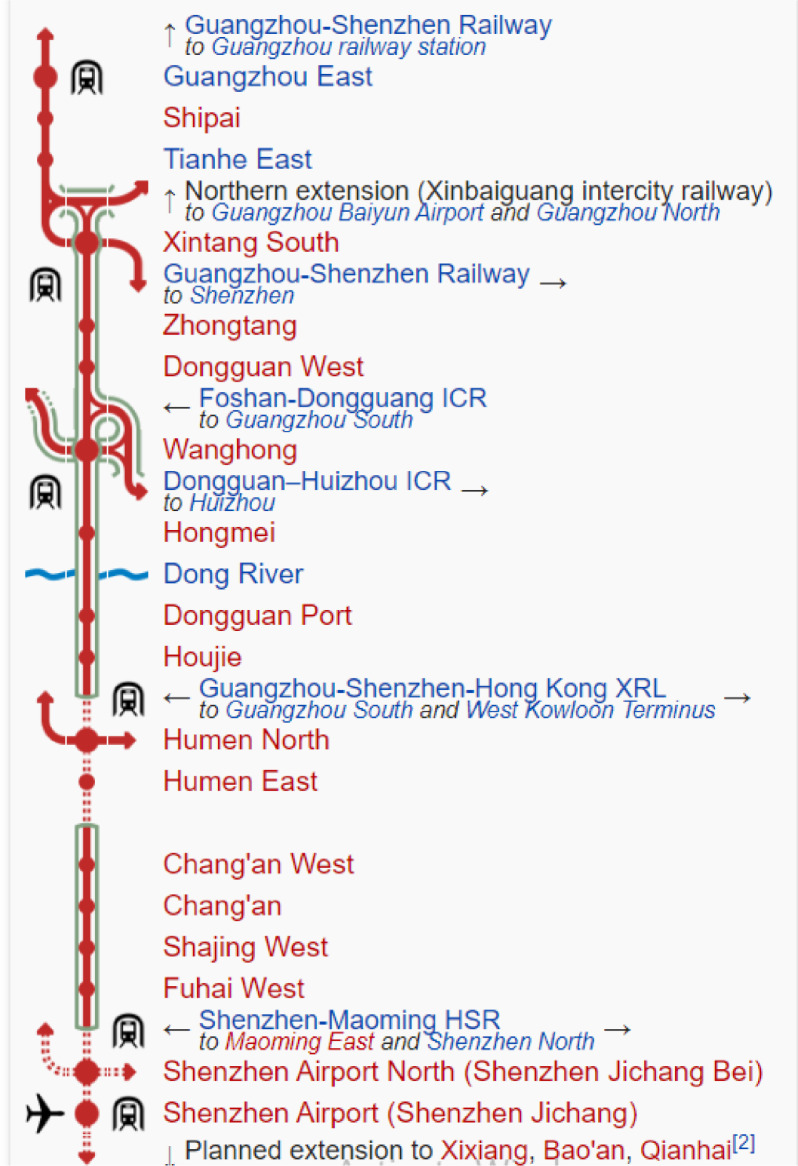
Guangzhou–Shenzhen intercity railway project [3].

The Guangzhou–Shenzhen intercity railway project is one of the greatest infrastructure projects in recent years (see Fig. 2) [3]. This project is located on the coast of the Pearl River Delta of Guangdong, China. The overall length of the project is about 116 km long and includes tunnels of a total length of 22 km. The construction project connects Guangzhou North Station and Bao'an International Airport, Shenzhen. The tunnel section is located in the zone of airport terminal 3, between Bao'an Airport North Station and Bao'an Airport Station. To construct the tunnel, an earth pressure balance shield machine is used [4], [5], [6]. The cutterhead shield machine is 8.85 m in diameter, and the trailing shield is 8.50 m in diameter, thereby leading to an over-cut annulus of 35.0 mm. The specifications of the earth pressure balance shield machine are listed in the original publication [1]. The main geological formation that encountered during tunnelling are silt clay and weathered rock. Table 2 lists the statistical analyses of the proposed model using different settings.

**Table 2:** Statistical results for different setting of proposed GMDH-GA model.

Model	Architecture				R^2^	RMSE
GMDH-GA	Mutation	Crossover	Generation	Population		
A	0.01	0.75	50	20	0.943	116.14
B	0.01	0.80	75	40	0.954	110.87
C	0.01	0.85	100	60	0.959	101.2
D	0.01	0.90	200	80	0.961	97.0
E	0.01	0.95	300	100	0.967	97.22

## 2 Experimental Design, Materials and Methods

To predict the disc cutter life, an intelligent model-a hybrid approach based on integrating group method of data handling (GMDH) with genetic algorithm (GA) was adopted [1]. This model is different from both traditional numerical models [7] and other artificial intelligence models, e.g. the gated recurrent unit (GRU) [6], evolutionary neural network [8], [9], [10], [11], [12], and long short-term memory (LSTM) [13]. The observed data in this article can be used to identify the applicability of the proposed model in which the collected data are divided into training set (22 variable) and testing set (10 variable). Table 2 lists the statistical results of the proposed GMDH-GA model. Regarding the application of the proposed GMDH-GA, different architectures were tested (Table 2), and an increase in the correlation coefficient (R^2^) and a decrease in the root mean square error (RMSE) was observed when the number of population of individuals and number of generations were increased. The best estimates were obtained using a network with double hidden GMDH layers. Hence, using the observation of the model number (E) in Table 2, the disc cutter life can be predicted in an appropriate manner.

## Declaration of Competing Interest

The authors declare that they have no known competing financial interests or personal relationships that could have appeared to influence the work reported in this paper.
